# Applying Ultrashort Pulsed Direct Laser Interference Patterning for Functional Surfaces

**DOI:** 10.1038/s41598-020-60592-4

**Published:** 2020-02-27

**Authors:** Daniel Wyn Müller, Tobias Fox, Philipp G. Grützmacher, Sebastian Suarez, Frank Mücklich

**Affiliations:** Chair of Functional Materials, Department of Materials Science, Saarland University, 66123 Saarbrücken, Germany

**Keywords:** Phase transitions and critical phenomena, Surfaces, interfaces and thin films, Surface patterning, Computational methods

## Abstract

Surface structures in the micro- and nanometre length scale exert a major influence on performance and functionality for many specialized applications in surface engineering. However, they are often limited to certain pattern scales and materials, depending on which processing technique is used. Likewise, the morphology of the topography is in complex relation to the utilized processing methodology. In this study, the generation of hierarchical surface structures in the micro- as well as the sub-micrometre scale was achieved on ceramic, polymer and metallic materials by utilizing Ultrashort Pulsed Direct Laser Interference Patterning (USP-DLIP). The morphologies of the generated patterns where examined in relation to the unique physical interaction of each material with ultrashort pulsed laser irradiation. In this context, the pattern formation on copper, CuZn37 brass and AISI 304 stainless steel was investigated in detail by means of a combination of experiment and simulation to understand the individual thermal interactions involved in USP-DLIP processing. Thereby, the pattern’s hierarchical topography could be tailored besides achieving higher process control in the production of patterns in the sub-µm range by USP-DLIP.

## Introduction

Highly specific surface properties in nature, like the well-known lotus and shark skin effects, are closely related to surface structures in the micrometre and nanometre length scale, often involving hierarchical patterns^1^. In fact, such biomimetic surface patterns have already proven to provide unique properties in several technical systems including the manipulation of contact mechanics and optical properties like light diffraction and absorption^2–4^. Patterns on the threshold between the micro- and the nanometre scale also showed to provide promising surface properties for medical products, as they can tailor the adhesion of both, cells and germs^5–7^. In this context, current research projects investigate the intricacies to prevent biofilm formation by surface patterning of different solid materials on the International Space Station (ISS), which endanger its crew in terms of both, health and damage to critical components^8^.

The predominant impact on the unique properties of patterned surfaces is defined by the scale and morphology of the surface features also including sub-patterns, which have to be adjusted specifically for each application. For instance, in antibacterial applications, feature sizes in the sub-µm length scale showed to be most effective against bacterial adhesion^7–10^. The processing methodology, dealing with such delicate surface modification, needs to ensure high machining precision as well as processing speeds and costs, which are able to compete with the classical methods of surface engineering. Besides lithographic methods, laser interference-based techniques have proven their worth in generating precise surface patterns, as they provide high processing speeds with little to no need for preparation and post-processing^11^. Direct Laser Interference Patterning (DLIP) using short pulsed laser sources was successfully applied for the controlled creation of periodic surface patterns in the micrometre scale, but has struggled to obtain sub-µm patterns on conductive materials^3–7,12^. Applying pulse durations close to or below the threshold of 10 ps in DLIP (which defines the ultrashort regime), allows for the generation of smaller pattern periodicities on a wider set of materials, even reaching below the µm scale range on metals^12–16^. When using ultrashort laser pulses (USP), sub-µm patterns can also be applied on several metals, semi-conductors and polymers by the formation of Laser Induced Periodic Sub-Structures (LIPSS)^10,12,17–19^. LIPSS generation is assumed to originate from the superposition of the incident laser pulse and a superficial plasmon wave within the irradiated substrate, inducing line-like sub-structures, which are oriented perpendicular to the laser pulse polarisation and exhibit a periodicity close to the laser wavelength. In addition to their use as primary pattern, LIPSS might be used as additional sub-patterns, rendering the morphology of DLIP generated patterns hierarchical^16^. Avoiding their formation, on the other hand, is often hard to achieve, especially on low conductive metals and semi-conductors. Hence, pattern morphologies on different materials generated by USP-DLIP might vary strongly in relation to the physical kinetics involved in topography formation, which need to be understood to efficiently tailor surface functionalities.

The current study investigates the material specific interaction in response to USP-DLIP for the creation of hierarchical structures in the micro- and sub-micrometre scale on ceramic, polymer and metallic materials involving primary pattern formation as well as sub-pattern generation. Since metals showed the most intricate response to processing by USP-DLIP in the experiments, the complex thermal surface kinetics involved in the pattern formation were examined by a combination of experiment and thermal simulation using a Two Temperature Model (TTM). Thus, the thermal and kinetic response of the noble metal copper, as well as the alloys CuZn37 brass and AISI 304 stainless steel on USP-DLIP could be determined in relation to a range of laser fluences and pulse accumulation. The knowledge of the material´s response allows for precise tailoring of the pattern’s topographical parameters and the creation of optimised patterns in the sub-µm scale.

## Methods

### Ultrashort pulsed direct laser interference patterning (USP-DLIP)

**Figure 1:**
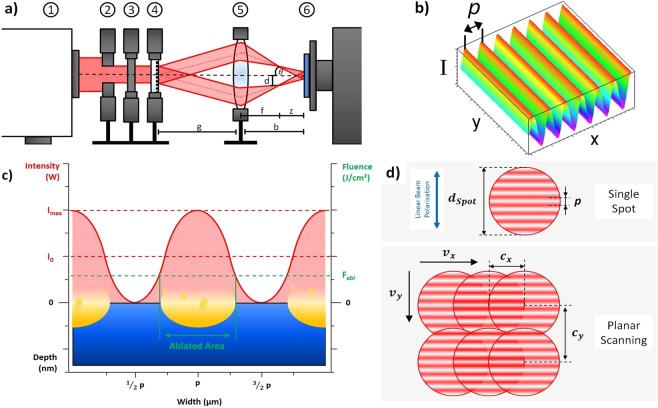
(**a**) Schematic illustration of the optical setup for USP-DLIP. 1 laser source, 2 aperture, 3 wave plate, 4 DOE, 5 lens systeme, 6 automated two axes (x, y) sample mount. (**b**) two beam interference leading to one-dimensional sinusodial intensity patterns. Periodicity *p* accounts for the distance between two intensity maxima. (**c**) Surface patterning takes place in the areas, where the laser fluence surpasses the material specific ablation threshold *F*_*abl*_. Hence, the pattern parameters can be modified by adjusting *p* and the seed beam intensity *I*_0_. (**d**) Single spot and planar scanning used for surface patterning also showing the alignment of the linear beam polarisation perpendicular to the pattern orientation. The overlap *c*_*x*_ and *c*_*y*_ were modified by adjusting the horizontal scanning speed *v*_*x*_ and the vertical step size *v*_*y*_ in relation to the pulse frequency of 1 kHz, the laser spot diameter *d*_*Spot*_ and the intensity pattern periodicity *p*.

For Direct Laser Interference Patterning a *Ti:Sapphire* laser source emitting ultrashort laser pulses with a pulse duration *t*_*p*_ of 100 fs at Full Width Half Maximum (FWHM) and a centred wavelength *λ* of 800 nm was used. In the optical setup, the seed beam first passes an aperture defining the working beam diameter, as well as altering the intensity profile from Gaussian to a near Top Hat. A wave plate adjusts the polarization angle of the laser beam perpendicular to the generated pattern orientation. The femtosecond pulsed seed beam is divided by a Diffractive Optical Element (DOE), while a lens system causes the coherent beams to overlap on the sample surface. Due to the short coherence length of approx. 30 µm in case of 100 fs laser pulses, deflection of the beam paths is kept as low as possible to still allow for interference across the full spot diameter. The optical setup is schematically shown in Fig. 1a. Overlapping the coherent beams induces an interference pattern modulating the distribution of the laser intensity on the substrate surface. In case of the two-beam interference setup used in these experiments, the pattern shows a one-dimensional sinusoidal distribution of laser intensity illustrated in Fig. 1b.

In the used optical setup, the individual beams are focussed before reaching the substrate’s surface. Thus, the interference zone is situated further along the beam path, where both beam diameters are widening again, which enables a faster processing due to the increased spot diameter. The analogy of the beam paths in the interference setup to the setup of the Fresnel mirror test can be utilised to calculate the periodicity *p* of the generated pattern in correlation to the incident angle *θ* of a single beam according to:

1 $$p=\frac{\lambda }{2\,\tan (\theta )}$$

The sinusoidal linear intensity distribution *I*(x) induced by DLIP can be calculated by^13^

2 $$I({\rm{x}})=2\,{I}_{0}\,co{s}^{2}(\frac{\pi {\rm{x}}}{p}+1)$$

Patterning of the irradiated substrate happens by physical laser-material-interaction, which depends on the local intensity gradient, as illustrated in Fig. 1c. In this context, the material response mainly corresponds to the material specific ablation threshold *F*_*abl*_^20^.

### Materials preparation

In the experiments different series of spots, as well as planar patterns with a periodicity *p* of 3 µm were applied on 10 × 25 mm platelets of the three metallic substrates copper, CuZn37 brass (*Wieland*) and AISI 304 stainless steel (*Brio*). The two ceramic and polymer samples, 3Y-TZP zirconia and Poly-L-Lactide Acid (PLLA), were solely planar patterned, both with 3 µm and 10 µm periodicity. The metallic substrates were first mirror-polished utilising an automated *TegraPol* system (*Struers*). Additionally, patterns of 0.7 µm periodicity were fabricated to investigate the possibility of producing sub-µm periodic structures on metallic substrates by this technique. To achieve this, the focal length of the lens system was changed from 100 mm (*p* = 3 µm) to 23 mm (*p* = 0.7 µm) by introducing an additional lens. The single spot samples were irradiated with different pulse numbers (n = 1, 2 and 5) at different laser fluences. Planar patterning was conducted by scanning the substrate surface in continuous pulsing mode using a pulsing frequency of 1 kHz. The different processing types are schematically shown in Fig. 1d. The overlap of the sequential pulses was tailored by the scanning speed, as well as the vertical spacing of the scanning lines as described in^21^. The laser fluence was altered from 0.5 to 4.6 J/cm² for *p* = 3 μm and from 2.07 to 7.6 J/cm² for *p* = 0.7 µm corresponding to the intensity calculated at FWHM of the DLIP-induced intensity pattern.

### Characterisation

Characterisation of the surface topography as well as the modification of the microstructure was conducted by means of confocal laser scanning microscopy (CLSM) utilising a *LEXT OLS4100 3D Measuring Laser Microscope* by *Olympus* as well as scanning electron microscopy (SEM) (*Helios 600* by *FEI*). The CLSM measurements were done using the 50× lens in digitally doubled magnification mode at a laser wavelength of 405 nm. For SEM imaging, secondary electron (SE) contrast was used in addition to a sample tilt of 52° degree, which allows for an improved visualization of topographical features. The acceleration voltage was set to either 10 kV or 5 kV at a current of 0.86 pA.

### Modelling the thermal response of metals to USP-DLIP

In case of an irradiation of metals by ultrashort laser pulses, the pulse duration and hence the time needed for a complete energy transfer to the material´s electron system is generally far shorter than the electron-phonon relaxation time^22^. This induces a high thermal gradient between electron gas and lattice leading to a very localized, strong heating. To predict the materials response correctly under these circumstances, the temperatures of both the electron system *T*_*e*_ and the lattice system *T*_*l*_ have to be considered separately in a Two Temperature Model (TTM)^23^. Corresponding to Fourier´s law of heating, the one dimensional distribution of *T*_*e*_ and *T*_*l*_ oriented orthogonal to the substrate surface is defined by the following equations^23,24^.

3 $${C}_{e}({T}_{e})\frac{\partial {T}_{e}}{\partial t}=[{k}_{e}({T}_{e})\frac{\partial {T}_{e}}{\partial z}]-G({T}_{e}-{T}_{l})+S$$

4 $${C}_{l}({T}_{l})\frac{\partial {T}_{l}}{\partial t}=[{k}_{l}({T}_{l})\frac{\partial {T}_{l}}{\partial z}]+G({T}_{e}-{T}_{l})$$

The parameters *C*_*e*_ and *C*_*l*_ represent the specific heat capacity of the electron and the lattice sub-systems, while *k*_*e*_ and *k*_*l*_ are their thermal conductivities. The energy transfer rate between electron gas and lattice is defined by the electron-phonon coupling coefficient *G*. The heat input induced by laser irradiation is represented by the source term *S*.

The electron heat capacity *C*_*e*_ of a Fermi-distributed electron gas is increasing linearly in relation to the electron temperature *T*_*e*_ for temperatures below the Fermi Temperature *T*_*F*_ = *E*_*F*_/*k*_*B*_. Hence, *C*_*e*_ can be calculated by *C*_*e*_ = *C*_*e*0_*T*_*e*_ where *C*_*e*0_ is the specific electron heat capacity at ambient temperature. For electron temperatures close to and higher than *T*_*F*_, the specific electron heat capacity should be determined by

5 $${C}_{e}=\frac{{N}_{e}{k}_{B}}{2}$$

where *N*_*e*_ is the electron density^24^.

The heat capacity *C*_*l*_ of the lattice can be estimated as being invariable for temperatures higher than the Debye temperature and hence represents a constant material parameter^25^.

Thermal diffusion in the electron system strongly depends on the local electron and lattice temperatures. For electron temperatures below *T*_*F*_, the thermal conductivity *k*_*e*_ of the electron system is suggested to follow the ratio between *T*_*e*_ and *T*_*l*_ as *k*_*e*_ ≈ *k*_*e*0_(*T*_*e*_/*T*_*l*_), where *k*_*e*0_ is the electron thermal conductivity at ambient temperature^26^. However, recent investigations have shown a more delicate behaviour of the electron thermal conductivity over a wider range of temperature, suggesting an improved term to determine *k*_*e*_ using the specific material constants *A* and *B*^27,28^.

6 $${k}_{e}={k}_{e0}\frac{B{T}_{e}}{A{T}_{e}^{2}+B{T}_{l}}$$

Nevertheless, by using Eq. (6) the electron thermal conductivity gets underestimated, when the electron temperature reaches a temperature much higher than the lattice temperature (by two orders of magnitude)^13,29^. This is most likely the case at high laser fluences and for metals exhibiting a low electron-phonon-coupling, like gold, silver and copper. Here, the electron thermal conductivity is represented more accurately by

7 $${k}_{e}=C\frac{{({\theta }_{e}^{2}+0.16)}^{5/4}\,({\theta }_{e}^{2}+0.44){\theta }_{e}}{{({\theta }_{e}^{2}+0.092)}^{1/2}\,({\theta }_{e}^{2}+s{\theta }_{l})}$$

where *θ*_*e*_ = *T*_*e*_/*T*_*F*_ and *θ*_*l*_ = *T*_*l*_/*T*_*F*_. The two material specific constants *C* and *s* can be determined by adjusting the progression of *k*_*e*_ to the behaviour calculated by Eq. (6) at low electron temperature, as described in^27^. In the simulations, Eq. (7) was preferred for copper and CuZn37, since the calculated values for *T*_*e*_ tend to significantly exceed *T*_*l*_ at higher fluences. In the case of steel, Eq. (6) was applicable for all the calculations, as the difference between *T*_*e*_ and *T*_*l*_ remained lower, which can be addressed to higher electron-phonon coupling.

As heat diffusion in metals is related up to 99% to transmissions by collisions within the electron system the contribution of *k*_*l*_ to the thermal diffusion can be neglected^30^.

Similar to the electron thermal conductivity, the electron-phonon coupling coefficient *G* depends on the system temperatures. It can be determined using the following equation^31^

8 $$G={G}_{0}[\frac{A}{B}({T}_{e}-{T}_{l})+1]$$

*G*_0_ is defined here as the electron-phonon coupling at ambient temperature.

The temporal intensity distribution of the laser pulse is following a Gaussian behaviour, defined by the pulse duration *t*_*p*_ at FWHM^32^. In conjunction to the Beer-Lambert Law and the pattern-shaped distribution of *I*(x) on the target surface defined by Eq. (2), the energy absorption of the irradiated substrate accounting for the source term is defined by^13^

9 $$S({\rm{x}},{\rm{z}},{\rm{t}})={\Delta }_{abs}\,\alpha (1-R)\frac{I(x)}{\sigma \,\sqrt{2\pi }}\exp [-\frac{{(t-{t}_{0})}^{2}}{2{\sigma }^{2}}-\alpha z]$$

In this term, *t*_0_ is the arrival time of the pulse at maximal intensity and *σ* the standard deviation defined by

10 $$\sigma =\frac{{t}_{p}}{2\,\sqrt{2ln2}}$$

The material specific absorptivity *α* and reflectivity *R* depend on the wavelength of the laser used to irradiate the substrate and can be calculated using the corresponding refractive index *n* and extinction coefficient *k*^33^ by

11 $$\alpha =\frac{4\pi k}{\lambda }$$

and

12 $$R=\frac{[{(n-1)}^{2}+{k}^{2}]}{[{(n+1)}^{2}+{k}^{2}]}$$

As former works have shown, the optical substrate properties are significantly changed due to strong thermal electron excitation during ultrashort pulsed laser irradiation, which increases the absorption of the laser pulse during irradiation^34–36^. To include this optical behaviour in the simulation of ultrashort pulsed laser-material interaction, *n* and *k* were altered according to enhanced electron energy states Δ*eV* using the data provided for copper and iron in^33^. Alteration of the optical properties was linked to the electron temperature *T*_*e*_ by approximating Δ*T*_*e*_ ~ Δ*eV*/*k*_*B*_.

Aside of the electron temperature, chemical or topographical surface modifications like oxidation or laser induced surface roughening also showed to enhance laser absorption of the substrate surface. In order to include this influence in the numerical calculation the factor Δ_*abs*_ was implemented in Eq. (9), which is raised from 1 to 2 according to the findings of Vorobyev *et al*.^37^ for the calculation of thermal interactions in response to multi pulse irradiation.

The thermal substrate response upon irradiation by USP-DLIP was analysed numerically in a 2D depth profile by solving the introduced thermal model with the simulation software *FlexPDE*. The specific material constants used in the numerical analysis of the thermal behaviour of copper and AISI 304 stainless steel are listed in Table 1. For stainless steel, the optical and thermal material parameters of iron were used, only adapting the material specific constants *A* and *B*, as well as the electron thermal conductivity *k*_*e*0_ to represent the low thermal conductivity of steel^38^. In the case of CuZn37 brass, the main parameters of copper were used, as its characteristics still dominate the metallic properties of this alloy. To specify the model for the alloy´s thermal behaviour, the lower thermal conductivity and heat capacity were implemented by choosing *k*_*e*0_ as 120 W/m³K and *C*_*l*_ as 3.17 × 10^6^ J/m³K.

**Table 1:** Specific material constants used in the TTM simulation of copper and stainless steel.

	Copper	Stainless steel
Absorptivity ${\alpha }_{(\lambda =800nm,Te=300K)}\,(\frac{1}{m})$^33^	12.8 × 10^7^	5.74 × 10^7^
Reflectivity ${R}_{(\lambda =800nm,Te=300K)}$^33^	0.9645	0.601
Electron heat capacity ${C}_{e0}(\frac{J}{{m}^{3}{K}^{2}})$^13,47^	96.6	706.4
Lattice heat capacity ${C}_{l}(\frac{J}{{m}^{3}{K}^{2}})$^48^	3.39 × 10^6^	3.66 × 10^6^
Electron thermal conductivity ${k}_{e0}(\frac{W}{mK})$^49,50^	401	15
Electron-phonon coupling coefficient ${G}_{0}(\frac{W}{{m}^{3}K})$^49^	12 × 10^16^	130 × 10^16^
Material specific constants C and $A(\frac{1}{sK})$^13,38^	*C* = 386.5	*A* = 0.98 × 10^7^
Material specific constants s and $B(\frac{1}{s{K}^{2}})$^13,38^	*s* = 0.14	*B* = 2.8 × 10^11^

## Results

### USP-DLIP on insulating materials

**Figure 2:**
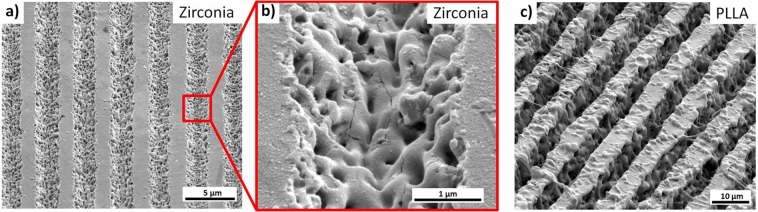
SEM-images of line patterns created by USP-DLIP on (**a**) 3Y-TZP zirconia at periodicity *p* of 3 µm and a fluence of 2.4 J/cm² as well as (**c**) PLLA at periodicity *p* of 10 µm and a fluence of 0.58 J/cm². (**b**) close-up of the ablation area on 3Y-TZP zirconia exhibiting porosity and cracks in the center, where the highest laser intensity was affecting the substrate surface.

For USP-DLIP on the insulating materials zirconia and PLLA *I*_0_ was adjusted accordingly to ablate an area of 50% of the whole spot area. To obtain continuous line patterns, the substrate surfaces were patterned with a pulse overlap *c*_*x*_ of 90%. The ablation areas on both materials are well defined and exhibit sharp boundaries to the non-ablated areas. The morphology of the ablated areas indicates that in both cases a liquid phase appears to have been present aside of predominant evaporative ablation during thermal interaction in response to USP laser irradiation. In the case of zirconia, the surface in the ablation area exhibits a high porosity accompanied by crack formation illustrated in Fig. 2a,b, which is likely to be induced by thermal stress from fast temperature transitions during laser processing. Similar observations were made on zirconia substrates processed by short pulsed DLIP, in case of a higher number of overlapped pulses^39^. The underlying mechanism leading to the occurrence of porosity could not be unambiguously determined, but it is most likely related to localized explosive vaporization events, which might originate from both inhomogeneity in the bulk material and enhanced localized absorption by laser induced surface roughening^37^. On PLLA the unaffected surface partitions exhibit spots and strands of re-solidified substrate that stretch across the ablation areas, as can be seen in Fig. 2c. In the experiments, remarkably high ablation depths of up to 10 µm were achieved in comparison to the other materials investigated at comparable processing parameters. Since neither zirconia nor PLLA have a band gap that matches the wavelength of the laser radiation used, electron excitation by multiphoton absorption is assumed, as also formerly observed for other ceramics and polymers^14,40^. This absorption mechanism was suggested to be able to induce avalanche ionisation, which is strongly increasing the absorptivity of the substrate in case of sufficiently high irradiation intensities^41^. This might be the reason for the high ablation depths observed for USP-DLIP on PLLA. As thermal interaction for both materials only occurs in case of irradiation intensities surpassing the critical threshold of multiphoton absorption, the sharp borderline between ablated and unimpaired substrate also speaks in favour of the postulated mechanism^29^. The laser fluence at FWHM surpassing the critical ablation threshold for multi photon absorption (leading to an ablation area of 50% of the periodicity *p*) accounts to 2.4 J/cm² for 3Y-TZP zirconia and 0.58 J/cm² for PLLA. Roitero *et al*.^39^ measured a similar ablation threshold *F*_*abl*_ for 3Y-TZP in case of short pulse durations of 10 ns, although melt formation clearly dominated the thermal interaction of the material. The comparable ablation thresholds despite the different pulse durations can be traced back to the lower laser wavelength of 355 nm used by Roitero *et al*., which appear to be energetically closer to the existing band gap of the material.

### USP-DLIP on metallic materials

**Figure 3:**
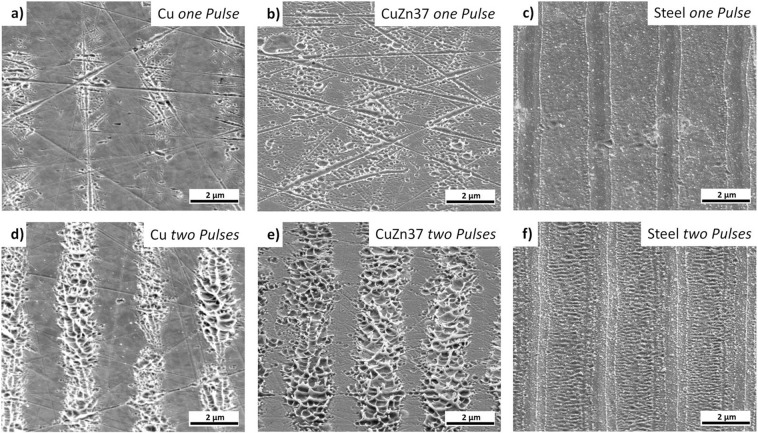
SEM-images of line patterns of 3 µm periodicity applied by USP-DLIP showing the different physical interaction of the three metallic substrates induced by USP-DLIP using a fluence of 1.1 J/cm², which corresponds to the single shot ablation threshold of copper for the utilised laser parameters^42^. (**a**) copper, (**b**) CuZn37, and (**c**) stainless steel after single pulse, (**d**) copper, (**e**) CuZn37, and (**f**) stainless steel after two pulses.

Examination of the surface topographies on both copper and CuZn37 induced by single pulse USP-DLIP at a fluence of 1.1 J/cm², which is close to the single pulse ablation threshold of copper^42^, reveals a morphology formed by superficial melting, as shown in Fig. 3a,b. In the proximity of surface scratches, single craters indicate localised vaporisation events. The craters appear more often and exhibit greater diameters on CuZn37. After dual pulse processing, the ablation rate and surface morphology on both materials is changed significantly. The roughened surface of a strongly agitated liquid phase, illustrated in Fig. 3d,e, is characterised by large craters, which appear to be initiated by explosive removal of molten matter. The affected area on both materials exhibits a significantly increased width in comparison to single pulse patterns, while patterns on copper show slimmer ablation areas in comparison to CuZn37 patterns. Both the extended width of the ablation area as well as the more frequent occurrence of localised vaporisation on CuZn37 might be linked to a reduction of the ablation threshold *F*_*abl*_ by alloying.

In comparison to single pulse processing, the ablation of material is significantly enhanced in dual pulsing. In fact, it was shown that consecutive pulsing alters the absorptivity of metals by oxidation as well as surface roughening, leading to multiple reflections and absorption of the irradiating laser beam^20,42^. Surface roughness in the nanometre-scale has been proven to effectively enhance the absorptivity up to two-fold^37^, which might also be the reason for enhanced thermal interaction at scratches on copper and CuZn37 surfaces, leading to the evaporation induced craters visible in Fig. 3a,b.

The topographies created on stainless steel by USP-DLIP strongly differ in morphology compared to the copper-based metallic substrates, as shown in Fig. 3c,f. The thermally affected area is marked by a clear melt-front. The smooth surfaces resulting from single pulse irradiation are replaced by a slight ripple formation after dual pulse irradiation. Although the pattern morphology does not indicate evaporation, ablation can be detected even for the lowest tested fluence of 0.5 J/cm², which is in good correlation to the USP induced ablation behaviour of iron measured by Artyukov *et al*.^43^. The ablation area width (i.e., line width) slightly increases after the second pulse. In the center of the ablation areas, ripple formation oriented perpendicular to the pattern orientation is visible. As the orientation of these ripples is parallel to the beam polarisation, they should not be related to LIPSS formation as it would be expected from other investigations^16,19^. The enhanced ablation of steel for the second pulse might be linked to higher absorption by laser induced oxidation and minor surface roughening due to the agglomeration of redeposited particles marked by a brighter SE contrast in Fig. 3c. Comparing the ablation area width between the illustrated DLIP patterns of the three metallic substrates in Fig. 3 additionally marks out the different ablation thresholds for single and multi-pulse irradiation.

### Thermal interaction of metallic substrates on USP-DLIP

**Figure 4:**
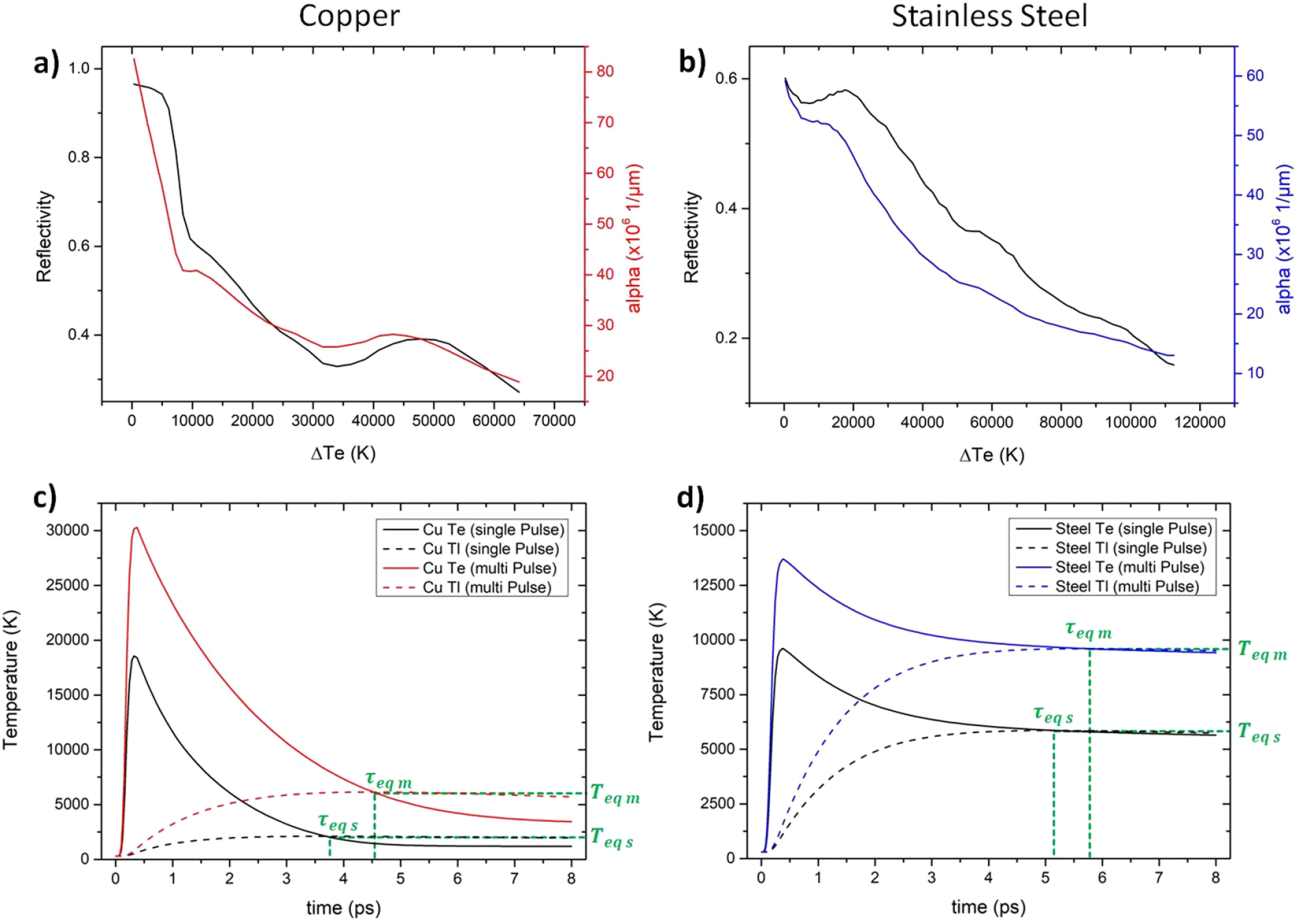
Alteration of the optical parameters of (**a**) copper and (**b**) stainless steel (literature data from iron) by enhanced electron temperatures during the laser pulse. Electron and lattice heating calculated for both (**c**) copper and (**d**) stainless steel at the surface area exposed to the maximum intensity of the DLIP pattern induced by a fluence of 1.1 J/cm² at single and multiple pulse irradiation. The thermal relaxation time *τ*_*eq*_ as well as the corresponding equilibrium temperature *T*_*eq*_ is marked for both single (*s*) and multi pulse (*m*) irradiation.

In order to study the thermal interaction of the different metallic substrates in response to USP-DLIP in detail, the substrate heating after irradiation by fluences of 0.5 to 4.6 J/cm² was simulated in 2D utilizing the aforementioned TTM. Alteration of surface absorptivity was involved in the calculation in two different ways: Modification of laser absorption due to excited electron states during laser irradiation was accounted for by modifying the optical parameters in relation to electron heating Δ*T*_*e*_, as mentioned in the Methods section. The approximated relation of reflectivity *R* and absorption coefficient *α* to Δ*T*_*e*_ are both shown in Fig. 4a,b. Experimental data achieved for fluence and equilibrium temperature dependent alteration of both parameters show good correlation to referenced experiments^34,36^. The enhanced absorptivity due to laser induced chemical and topographical surface modification was addressed by enhancing the value of the absorption factor Δ_*abs*_ in Eq. (9). According to the findings of Vorobyev *et al*.^37^, the chosen value of Δ_*abs*_ for the calculation of the thermal substrate interaction on consecutive pulses was 2, while it was kept 1 for single pulse irradiation. The change in thermal response of both copper and stainless steel by altered surface absorptivity is illustrated for an irradiation fluence of 1.1 J/cm² in Fig. 4c,d. Both graphs exhibit a significant rise in the resulting heating of both materials due to enhanced laser absorption, which especially is true for copper.

**Figure 5:**
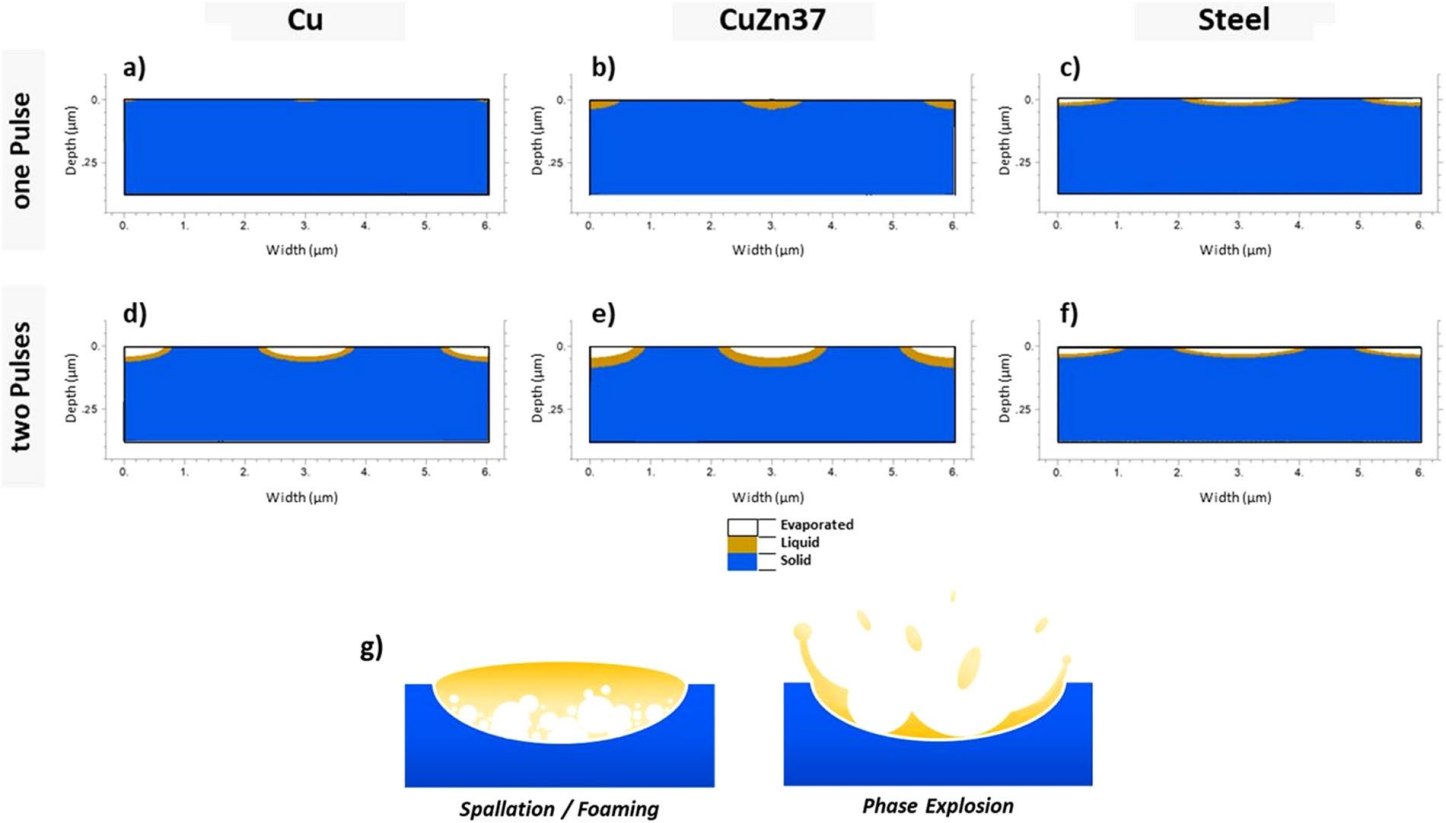
Numerical simulation plots of the phase changes in each of the three metallic substrates induced by lattice heating after single and dual pulse USP-DLIP at a fluence of 1.1 J/cm² and 3 µm periodicity. The utilised parameters correspond to the experimental setup used to fabricate the patterns illustrated in Fig. 3. (**a**) copper, (**b**) CuZn37, and (**c**) stainless steel after a single pulse, (**d**) copper, (**e**) CuZn37, and (**f**) stainless steel after two pulses. (**g**) Illustration of the two mechanisms of USP induced thermal ablation on highly conductive metals^46^, involving either expansion of subsurface vapour cavities (spallation) or explosive expansion of a superheated vapour-liquid mixture (phase explosion).

To estimate the lattice heating induced by USP-DLIP responsible for phase changes and material ablation, the 2D depth profile of the lattice temperature at thermal relaxation *τ*_*eq*_ is calculated, marking the highest lattice temperature. As electron-phonon-coupling transitions are assumed to have the main effect on lattice heating in the ultrashort time regime, the influence of heat diffusion in the lattice system after *τ*_*eq*_ was neglected^30^. Phase fronts were estimated by following the isothermal frontiers of the threshold temperatures for solid/liquid and liquid/vapour phase transitions. In the context of ultrafast phase transitions within hundreds of femtoseconds, Rethfeld *et al*.^29,44^ discussed the stimulation of homogeneous melting of metals and semiconducting materials due to strong electronic in-equilibria in the substrate surface induced by ultrashort laser pulses. In case of copper, the critical lattice temperature leading to homogeneous melting within a pulse duration of 100 fs is assumed as *T*_*hm* 100 *fs*_ = 1.45 × *T*_*m*_, with *T*_*m*_ as the melting temperature of copper^44^. Reduced metallic behaviour of the irradiated material was shown to decrease the threshold and hence the period between irradiation and formation of superheated liquid^44,45^. This effect appears to be triggered by a change in atomic bond forces after intense superficial electron emission^29,45^. According to this theory, threshold temperatures for ultrafast homogeneous melt formation during ultrashort pulsed irradiation of less conductive metals and alloys like CuZn37 and steel can be estimated to be in a considerable lower relation to *T*_*m*_ compared to copper^45^. Hence, the solid/liquid threshold temperature *T*_*sl*_ for copper was chosen at *T*_*sl*_ = 1.45 × *T*_*m*_, while in case of both alloys *T*_*sl*_ was approximated to be equal to *T*_*m*_. The liquid/vapour threshold temperature *T*_*lv*_ for all materials was estimated as the boiling temperature *T*_*b*_. The calculated distribution of the different material states at *τ*_*eq*_ after single pulse and dual pulse USP-DLIP irradiation at a fluence of 1.1 J/cm² is illustrated for the three different metallic substrates in Fig. 5a to f corresponding to the patterns displayed in Fig. 3. On both copper and CuZn37 the effect of enhanced surface absorptivity due to altered surface roughness and chemistry on thermal interaction clearly stands out due to the increase of both width and depth of the thermally affected area observed after two pulses. In both cases, the liquid/vapour threshold was not surpassed after single pulse irradiation emphasising the relation of crater formation by evaporation to locally enhanced surface roughness by scratches in Fig. 3a,b. The thermally affected area on stainless steel already exhibits a large width after single pulse irradiation accompanied by a comparably shallow depth, which both increase slightly after dual pulse irradiation. This behaviour might be traced back to both the comparably low surface reflectivity for the irradiating wavelength of 800 nm as well as the smaller change of both surface reflectivity and the absorption coefficient in the present range of electron heating. Thereby, enhanced absorptivity by laser induced surface modification has a less serious effect compared to copper.

The observed ablation of highly conductive metals like copper after ultrashort pulsed irradiation can be separated into two different mechanisms: At fluences slightly higher than the ablation threshold, ablation is linked to *spallation* (or *foaming*), which is denoting heterogeneous vapour nucleation in a subcritical liquid phase^43,46^. Spallation results in minor ablation, as measured after single pulse USP-DLIP. When the fluence significantly surpasses the ablation threshold, the material’s ablation is associated with evaporative supercritical fluid expansion, also denoted as *phase explosion*^46^. This mechanism is induced by an adiabatic unloading of a supercritical liquid phase leading to a strong impulse, detaching the vapour/liquid mixture from the surface at high velocities. The explosive removal of hot matter initiates a strong convective cooling effect, which is limiting further heat diffusion and freezes the agitated melt-front, leading to the characteristic topographies of churned up melt^43^. This corresponds well to the morphology visible on the surfaces treated by dual pulse USP-DLIP illustrated in Fig. 3d,e. In case of phase explosion, ablation is significantly enhanced as the kinetic impulse also removes a considerable amount of molten material, while for spallation localized boiling with minor melt mobilisation can be observed, as schematically shown in Fig. 5g.

**Figure 6:**
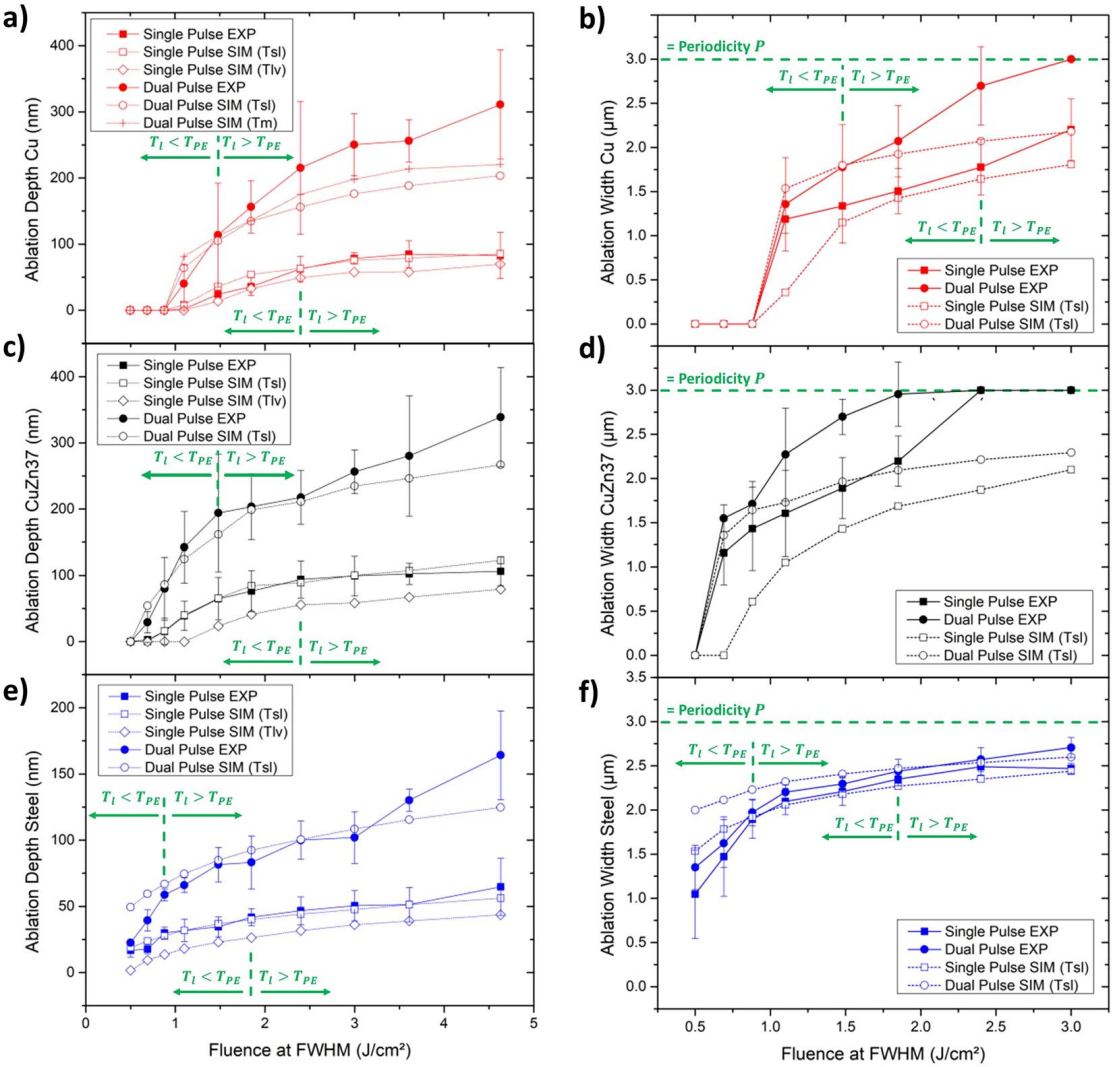
Comparison of the numerically calculated and measured (CLSM) ablation depth and width in relation to laser fluences in the range of 0.5 to 4.6 J/cm² after single and dual pulse USP-DLIP on the three metallic substrates: (**a**) ablation depth and (**b**) ablation area width on copper, (**c**) ablation depth and (**d**) ablation area width on CuZn37, (**e**) ablation depth and (**f**) ablation area width on stainless steel. The parameters, where the lattice temperature exceeds the phase explosion threshold temperature *T*_*l*_ > *T*_*PE*_ in the numerical calculations are additionally marked in (**a**–**c**,**e**,**f**).

Wang *et al*.^36^ linked the onset of phase explosion on copper to lattice heating surpassing a threshold temperature of *T*_*PE*_ = 0.9 × *T*_*cr*_ with a critical temperature *T*_*cr*_ of 7696 K. The relation of ablation depths measured by CLSM to the calculated melting and vaporisation depth for single pulses on copper at the different tested laser fluences, illustrated in Fig. 6a, shows a good correlation to the experimental results. Thereby, it can be noted that the measured ablation depth starts to match the calculated melting depth as soon as the maximum lattice temperature surpasses *T*_*PE*_. This can be traced back to melt expulsion expected during phase explosion. In case of dual pulse irradiation, *T*_*PE*_ is reached at significantly lower laser fluences due to the enhanced laser absorption. Here, experimental values for ablation induced by phase explosion exceed the calculated depth of melt formation for an estimated homogeneous melting threshold of *T*_*sl*_ = 1.45 × *T*_*m*_ for *T*_*l*_ > *T*_*PE*_. This also applies if the considered melting threshold is reduced to *T*_*m*_ of copper. This effect appears to be attributed to an accumulation of melt ejections at the edges of the ablation zone. In support of this theory, the experimentally measured ablation width tends to permanently exceed the calculated width of melt induction, as illustrated in Fig. 6b. It should be emphasised that the continuous increase in ablation width on copper leads to an overlap of the ablation areas after the second pulse for fluences higher than 2.4 J/cm². Hence, a complete covering of the substrate surface by expulsed melt can be assumed for multi-pulsed USP-DLIP substrate processing at a periodicity of 3 µm.

In case of CuZn37, the effect of a complete covering of the substrate surface by expulsed melt is already expected for a reduced threshold fluence of 1.88 J/cm² as a result of a lower ablation threshold as visible in Fig. 6d. Here, experimentally measured ablation depth shows a strong correlation to the calculated melting depth for single pulse irradiation without any indication of a change from ablation by spallation to phase explosion (see Fig. 6c), even though, ablation on CuZn37 appears to follow similar kinetics like on copper, judging from the resulting surface morphologies imaged by SEM. A possible reason for the contradictory results could be the stronger, scratch-induced crater formation after single pulses on CuZn37, which can lead to a higher average depth observed in the experiments. A topography-induced influence of the experimental values by scratches at low fluences can also be assumed by the strong correlation between measured single and dual pulse ablation width visible for both copper and CuZn37 in Fig. 6b,d, indicating similar effective ablation thresholds and leading to a strong deviation from the calculated ablation width at low fluences. In case of dual pulse irradiation, a transition from spallation to phase explosion might be assumed between 0.88 J/cm² and 1.1 J/cm² from the comparison of experimental and calculated values. However, this is questionable due to the ambiguous values of single pulse ablation. Neither single nor dual pulse ablation can be linked to the phase explosion threshold temperature *T*_*PE*_ of copper, indicating that other ablation mechanisms and/or threshold temperatures need to be considered for the USP thermal interaction for the alloy.

Tsibidis *et al*.^38^ indicate a similar relation of phase explosion to the critical temperature for steel as Wang *et al*.^36^ using the critical temperature of iron (*T*_*cr*_ = 8500 K) for their calculations. However, USP-DLIP patterns produced on stainless steel do not exhibit traces of spallation or phase explosion in our observations, which might be linked to the influence of superficial electron emission on atomic bond strength as discussed by Rethfeld *et al*.^29^. Accordingly, the resulting surface charging in the case of low-conductive steel might favour the formation of low-temperature plasma formation on the substrate surface during thermal interaction after USP irradiation. In this case, removal of superheated material can take place before spallation or phase explosion is induced. Thereby, thermal energy losses by plasma emission could cause a strong freezing effect on the electron subsystem, keeping the overall heating permanently below the threshold of spallation or phase explosion^46^. A similar behaviour was already reported for iron^43^. Consequently, patterns induced by single pulse USP-DLIP on stainless steel surfaces show a similar relation to the calculated melting depth as CuZn37, where changes between ablation mechanisms are not visible, as illustrated in Fig. 6e. Even though, phase explosion is not visible on steel, the melt seems to be displaced out of the ablation area during ablation, which is also indicated by the clear melt throw-ups marking the boundaries of the individual ablation areas in Fig. 3c,f. After dual pulse processing, on the other hand, measured ablation remains below the calculated melting depth until the theoretical phase explosion threshold estimated by Tsibidis *et al*.^38^ is surpassed. A similar approximation of the measured and calculated values can be observed for the ablation width of stainless steel in the low fluence regime, illustrated in Fig. 6f. However, the observed ablation behaviour is not equally connected to *T*_*PE*_ of iron for both single and dual pulse irradiation. Additionally, surface morphologies in both cases do not indicate phase explosion ablation, by which an actual involvement of this parameter in thermal interaction of steel on USP irradiation is questionable. Comparing the measured ablation depths after single pulse irradiation of copper and stainless steel to the experimental results of Wang *et al*.^36^ and Artyukov *et al*.^43^ exhibit a good correlation of our own findings to literature data for the investigated range of fluence.

**Figure 7:**
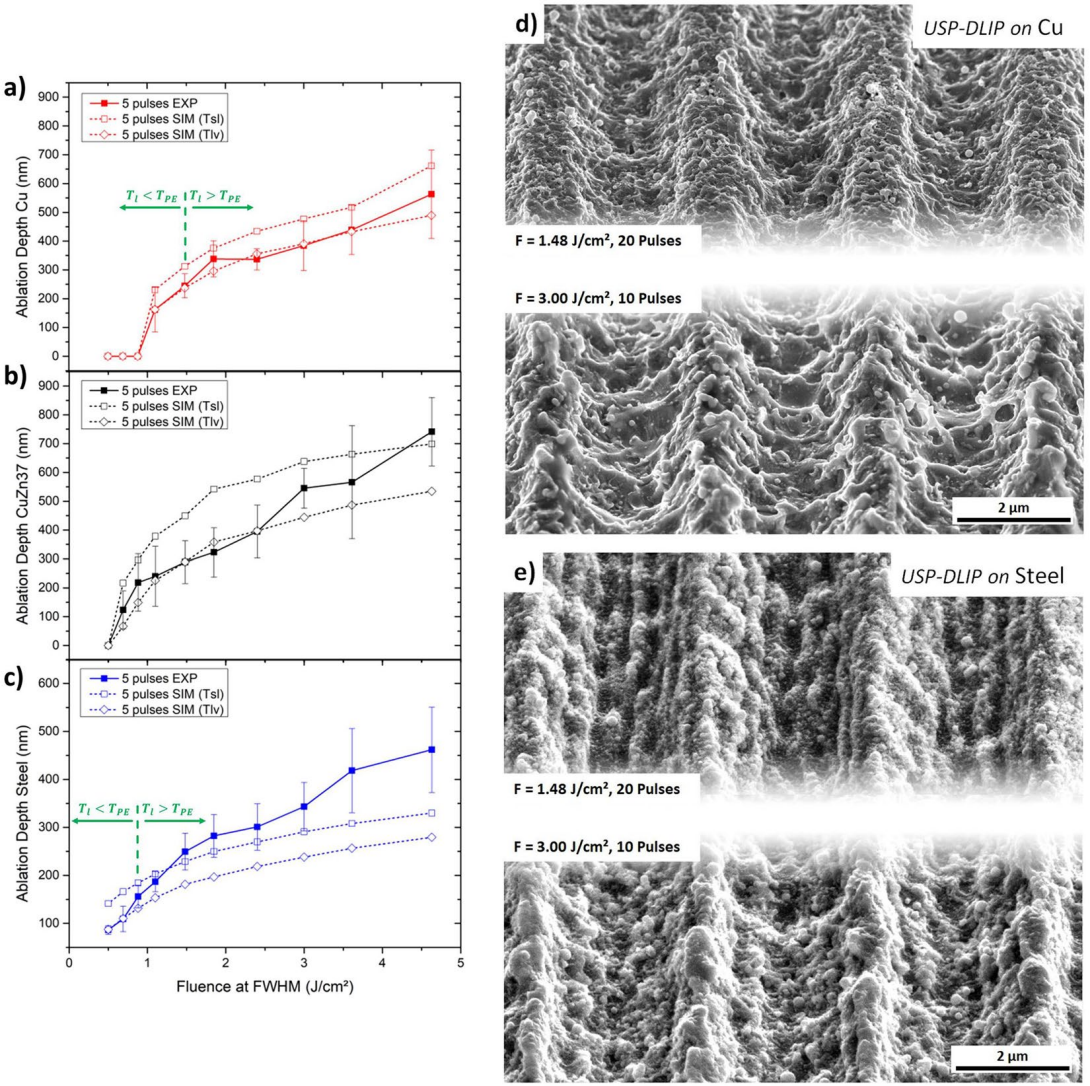
Ablation depth measured by CLSM on USP-DLIP patterns compared to simulated results of melting and vaporisation depth after irradiation by five consecutive pulses at fluences ranging from 0.5 to 4.6 J/cm² on the three metallic substrates (**a**) copper, (**b**) CuZn37 and (**c**) AISI 304 stainless steel. (**d**) Modification of the surface parameters on copper by adaption of the processing parameters in favour of faster processing speed leading to sharper pattern tips and a melt dominated sub-pattern. (**e**) Inhibiting LIPSS-formation on stainless steel by adaption of the processing parameters in favour of both higher laser intensity and lower pulse agglomeration.

Aside of the different mechanisms involved in ablation and surface morphology formation, all three metallic substrates exhibit a significant increase of the experimentally measured ablation depth in relation to the calculated melting depth at higher fluences, especially for dual pulse processing. This behaviour appears to be related to an increasing overlap of the ablation areas, leading to an accumulation of ejected melt between the ablation areas. For greater pulse numbers, this effect on pattern depth mostly vanishes in case of copper. Here, the ablated volume can be mainly related to evaporation by comparing measured and calculated ablation depth, illustrated in Fig. 7a. On CuZn37, ablation also appears to be related to evaporation for fluences below 2,4 J/cm², but yet exhibits an increasing effect of melt expulsion from 3 to 4.6 J/cm², similar to the additionally enhanced ablation depths measured after dual pulse irradiation, as can be seen in Fig. 7b. Although the surface of steel does not show any characteristics of phase explosion even after five pulses, ablation can again be linked closely to the threshold temperature *T*_*PE*_ of iron^38^. This is demonstrated by an increasing involvement of melt expulsion in ablation as soon as the lattice temperature surpasses this threshold, as illustrated in Fig. 7c. At a fluence of 1.48 J/cm², the measured ablation even surpasses the corresponding calculated melt depth, until the measured vales are almost twice as high as the calculated values at 4.6 J/cm². Here, USP-DLIP processing of stainless steel might be benefiting from the smaller expansion of the ablation area during consecutive pulsing in contrast to the enhanced expansion by melt expulsion during phase explosion on copper and CuZn37 (compare Fig. 6b,d,f).

### Effect of thermal interaction on surface morphology

The ablation mechanisms observed in the USP-DLIP processing of the different metallic substrates are likely to not only influence ablation, but also the surface morphology of the patterns. This involves the formation of sub-patterns like in particular LIPSS on stainless steel. In case of copper, the two observable ablation mechanisms spallation and phase explosion differ mainly in the amount of melt agitation during the ablation process. The comparison of patterns produced on copper by a fluence of 1.48 J/cm², where the threshold temperature *T*_*PE*_ for phase explosion at consecutive pulsing is barely reached in the simulations, and 3 J/cm², where *T*_*PE*_ is considerably surpassed, is illustrated in Fig. 7d. The aspect ratio of the patterns was adjusted to be equal by enhancing the pulse overlap for the lower fluence from 90% to 95%, leading to an increase in pulse number from 10 to 20 pulses. The difference in ablation width between the two laser parameters is clearly visible, leading to almost flat pattern peaks at lower fluence, while the peak sharpness at higher fluence is enhanced by the agglomeration of expulsed melt. Aside of the main pattern geometry, the morphology the sub-patterns also differ corresponding to the different ablation mechanisms. In case of 1.48 J/cm², spallation ablation leads to smaller scale feature sizes of the sub-pattern, which is dispersed equally on the whole surface. On patterns processed at a fluence of 3 J/cm², the sub-pattern is formed by re-solidified melt, which was agitated during phase explosion inducing both a bigger feature size of the sub-pattern as well as a difference in surface morphology between the pattern peaks and valleys.

On steel surfaces processed by USP laser irradiation, LIPSS formation dominates the sub-pattern morphology at a fluence of 1.48 J/cm², which is mainly related to an agglomeration of a higher number of pulses at low laser fluence^19,38^. Consequently, LIPSS formation is significantly reduced on stainless steel substrates by applying a lower pulse overlap combined with enhanced laser fluence, as visible in Fig. 7e. The parameters utilised for USP-DLIP processing of the displayed patterns on steel were the same as the ones used on copper. In case of steel, the difference in fluence is only recognizable by the different slopes at the flanks of the peaks, as the main pattern geometries resemble each other closely. Both patterns exhibit a high peak sharpness attributable to melt agglomeration between the ablation areas similar to the pattern formed on copper at higher laser fluence. This might be linked to the fact that in both cases, the threshold temperature *T*_*PE*_ was surpassed. As for the patterns produced on copper, similar aspect ratios were achieved by adjusting the pulse overlap accordingly to the alteration of the laser fluence.

**Figure 8:**
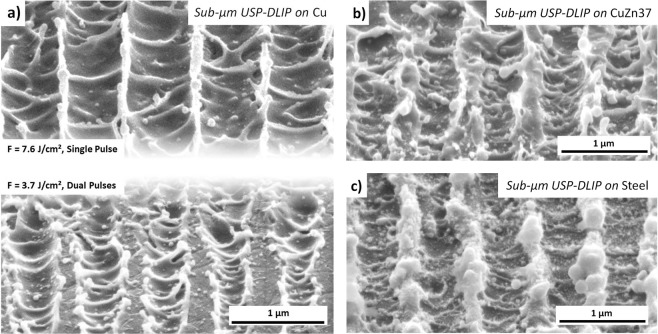
Display of sub-µm patterns on each of the investigated metallic substrates exhibiting a periodicity of 0.7 µm. (**a**) Comparison between sub-µm patterns on copper processed at a fluence of either 7.6 J/cm² or 3.6 J/cm², where pattern preservation was improved at lower fluence. (**b**) sub-µm pattern on CuZn37 processed at a fluence of 2.07 J/cm² by two consecutive pulses. (**c**) sub-µm pattern on AISI 304 stainless steel processed at a fluence of 6.1 J/cm² by two consecutive pulses.

In case of lower pattern periodicities, both enhanced absorptivity by laser induced surface modification as well as melt expulsion during ablation might play a crucial role in DLIP processing. Bieda *et al*.^13^ for example showed a critical enhanced absorptivity in DLIP processing of copper using near-USP durations, where sub-µm patterns could be applied by single pulses, but vanished after dual pulse irradiation. A similar behaviour was observed in our experiments, after irradiation of copper at a fluence of 7.6 J/cm², where the sub-µm pattern exhibits a significant overlap of the ablation areas already after single pulse irradiation. By reducing the fluence to 3.7 J/cm², a less critical surpassing of *T*_*PE*_ was achieved in dual pulse irradiation, whereby the pattern could be maintained for two consecutive pulses, as illustrated in Fig. 8a. Similar results for two pulses were achieved for CuZn37 at a fluence of 2.07 J/cm² and steel at a fluence of 6.1 J/cm², where the difference between the applicable fluences once again highlights the effect of the individual ablation behaviour on USP-DLIP processing of the different metals. The patterns on both alloys corresponding to the mentioned processing parameters are displayed in Fig. 8b,c.

## Conclusions

USP-DLIP processing at near-infrared wavelength of the three different material groups ceramics, polymers and metals was achieved using similar laser fluences. The processability of materials showing low absorptivity at this wavelength can be attributed to the altered laser-material interaction during USP irradiation. The influence of the ultrashort pulse duration on the thermal interaction was discussed. Special attention was paid to processability as well as to surface morphology for the individual material groups, pointing out the unique specifications, which need to be considered in USP-DLIP.

On insulating materials, ablation is strongly related to the critical threshold fluence inducing multiphoton absorption. Measurable ablation only happens when the laser fluence surpasses this threshold leading to sharply defined pattern features. Thereby, sub-pattern formation within the clear-cut ablation areas represents substrate specific thermal responses involving both melting and evaporation that lead to unique pattern morphologies. Alteration of the processing parameters appears to only influence the amount of ablation without impacting the morphology of the ablation area.

In case of metals, an alteration of the occurring ablation mechanism related to material specific threshold fluences has to be taken into account. At elevated fluences, which induce lattice heating considerably surpassing the material-specific phase explosion threshold temperature *T*_*PE*_, a strong influence of melt agitation on pattern formation has to be considered. Numerically, this also seems to apply to the pattern formation on steel surfaces, although no change in the ablation mechanism has been observed based on surface morphology. At low fluences, pattern geometries on copper are more defined (e.g. exhibiting higher edge sharpness) similar to USP-DLIP patterns on insulating materials, while at fluences inducing lattice heating above *T*_*PE*_ melt expulsion appears to dominate pattern formation. On AISI 304 stainless steel, lower fluences mainly result in enhanced LIPSS formation, which can consequently be reduced by increasing the laser fluence. Pattern geometry formation on steel is dominated by melt agitation for all parameters investigated, where it must be noted that the phase explosion threshold temperature *T*_*PE*_ of iron was surpassed in each case.

It was shown that laser induced surface roughening and oxidation plays a significant role in USP-DLIP of materials exhibiting low absorptivity for the utilised near-infrared wavelength, as it greatly increases the laser absorption. Thereby, significant enhancement of ablation, as well as a change in ablation mechanism has to be considered on highly conductive metals for consecutive pulsing. This has to be taken into account especially in the creation of sub-µm patterns, where the enhanced laser absorption might lead to a vanishing of the pattern by a single subsequent pulse.

In summary, we were able to show that both pattern geometry as well as hierarchical sub-pattern formation during USP-DLIP processing of different materials can be controlled by taking their individual thermal interaction into account. By this, surface functionalisation for manifold applications including e.g. wettability or bacterial adhesion can be tailored more precisely, thus enhancing the effect of the surface treatment.

## Data Availability

The experimental data generated in this work can be made available on request.
